# Error in Byline

**DOI:** 10.1001/jamanetworkopen.2026.8356

**Published:** 2026-03-24

In the Consensus Statement titled, “Guidelines for the Prevention, Diagnosis, and Management of Urinary Tract Infections in Pediatrics and Adults: A WikiGuidelines Group Consensus Statement,”^1^ published November 4, 2024, there was an error in the byline. Dr Abdul Azim’s surname should not have had a hyphen. This article has been corrected.^1^

## References

[1] Nelson Z, Aslan AT, Beahm NP, . Guidelines for the prevention, diagnosis, and management of urinary tract infections in pediatrics and adults: a WikiGuidelines group consensus statement. JAMA Netw Open. 2024;7(11):e2444495. doi:10.1001/jamanetworkopen.2024.44495 39495518

